# Nasopharyngeal Carriage and Antimicrobial Susceptibility Patterns of *Streptococcus pneumoniae* among Children under Five in Southwest Ethiopia

**DOI:** 10.3390/children4040027

**Published:** 2017-04-19

**Authors:** Tiglu Gebre, Mulualem Tadesse, Dossegnaw Aragaw, Dagne Feye, Habtamu Bedimo Beyene, Dinberu Seyoum, Mekidim Mekonnen

**Affiliations:** 1 Department of Medical Laboratory Sciences and Pathology, College of Health Sciences, Jimma University, P.O. Box 378, Jimma, Ethiopia; tigayde@gmail.com (T.G.); dos.aragaw@gmail.com (D.A.); mekidim.mekonnen@ju.edu.et (M.M.); 2 Mycobacteriology Research Center, Institute of Biotechnology, Jimma University, P.O. Box 378, Jimma, Ethiopia; 3 Department of Pediatric and Child Health, Shanan Gibe Hospital, P.O. Box 378, Jimma, Ethiopia; dagnefeye@gmail.com; 4 Department of Microbiology, Immunology and Parasitology, College of Health Sciences, Addis Ababa University, P. O. Box 9086, Addis Ababa, Ethiopia; habtish1976@gmail.com; 5 Department of Statistics, College of Natural Sciences, Jimma University, P.O. Box 378, Jimma, Ethiopia; dinberu.seyoum@gmail.com

**Keywords:** *Streptococcus pneumoniae*, nasopharyngeal carriage, antimicrobial susceptibility, risk factor

## Abstract

Nasopharyngeal carriage of *Streptococcus pneumoniae* is found to play an important role in the development and transmission of pneumococcal diseases. In this study, we assessed the nasopharyngeal carriage, antimicrobial susceptibility patterns and associated risk factors of *S. pneumoniae* among children under five. A total of 361 children under five attending the outpatient department of Shanan Gibe Hospital in Jimma, Southwest Ethiopia were enrolled from June to September 2014. Nasopharyngeal specimens were collected using sterile plastic applicator rayon tipped swab and inoculated on tryptone soy agar supplemented with 5% sheep blood and 5 µg/mL gentamycin. Antimicrobial susceptibility testing was performed using the modified disk diffusion method. The overall prevalence of *S. pneumoniae* carriage was 43.8% (158/361) among children under five. Resistance to tetracycline, cotrimoxazole, penicillin, chloramphenicol and erythromycin was observed in 53.2% (84/158), 43.7% (69/158), 36.1% (57/158), 13.3% (21/158) and 8.9% (14/158) of isolates respectively. Multidrug resistance was seen in 17.7% (28/158) of isolates. In multivariate logistic regression analysis, children living with sibling(s) < 5 years old (adjusted odds ratio (AOR) = 1.798; 95% confidence interval (CI), 1.169–2.766) and malnutrition (AOR = 2.065; 95% CI, 1.239–3.443) were significantly associated with *S. pneumoniae* carriage. A high nasopharyngeal carriage of *S. pneumoniae* was observed among children under five in Southwest Ethiopia. There should be a strategy to prevent *S. pneumoniae* nasopharyngeal colonization and identify the appropriate antibiotic to the individual child.

## 1. Introduction

*Streptococcus pneumoniae* (pneumococcus) is a major cause of childhood morbidity and mortality worldwide, particularly in lower income countries [1,2]. *S. pneumoniae* is the leading cause of bacterial pneumonia and meningitis [3]. It is responsible for 15–50% of all episodes of community acquired pneumonia, 30–50% of all cases of acute otitis media, and a significant proportion of bacteremia and bacterial meningitis [4]. *S. pneumoniae* is the most significant cause of bacterial pneumonia deaths and accounted for 33% of such deaths in 2010 in children under five years of age [5]. The World Health Organization (WHO) estimated that there are nearly 1 million deaths each year in children younger than five years of age and, a child under five years of age dies because of it every 20 s [6].

*S. pneumoniae* is the leading cause of pneumonia and the largest single infectious killer of children in Ethiopia [7]. Each year, approximately 57,000 of deaths are due to pneumococcal infections in children under five years of age [7,8]. In Ethiopia pneumococcal conjugate vaccine (PCV-10) has been introduced into the routine child immunization program since 2011. This is given to young children with the recommended three dose schedules at 6, 10 and 14 weeks of children age. Despite the introduction of PCV-10 for majority of children in Ethiopia, *S. pneumoniae* remains the major causes of mortality in under-fives, accounting for 21% of mortality in this group [9]. 

The nasopharynx is known to be the main ecological niche of *S. pneumoniae*, from where it can give rise to disease after extending to other areas of the respiratory tract or penetrating normally sterile body fluids [10]. *S. pneumoniae* nasopharyngeal (NP) carriage was found to play an important role in the development and transmission of invasive and noninvasive pneumococcal diseases [11,12]. Infants and young children are considered to be the main reservoir of *S. pneumoniae* [8].

Nasopharyngeal colonization by antimicrobial resistant *S. pneumoniae* has been increasing in different parts of the world including Ethiopia [13,14,15]. In resource-poor settings, therapy remains empiric because of the lack of rapid and accurate diagnostic tests. Moreover, antibiotic choices in Ethiopia are significantly governed by cost rather than effectiveness. Investigating the geographical variations in antimicrobial resistance and monitoring trends in antimicrobial resistance development are essential for adequate antibacterial therapy. There is an urgent need to investigate the nasopharyngeal carriage and specific trends of antimicrobial resistance in *S. pneumoniae* in different parts of Ethiopia. This study was, therefore, carried out to determine nasopharyngeal carriage rate, antimicrobial susceptibility pattern and possible risk factors of *S. pneumoniae* among children under five attending the outpatient department in Shanan Gibe Hospital, Southwest Ethiopia.

## 2. Material and Methods

### 2.1. Study Setting and Population

A cross-sectional prospective study was carried out between June and September 2014 in Shanan Gibe Hospital, a primary hospital in Jimma town, Southwest Ethiopia. According to the 2007 population and housing census of Ethiopia, the projected total population of Jimma town in 2014 was 169,446 (84,508 males and 84,938 females) [16]. The Shanan Gibe hospital provides curative and preventive services for more than 100,000 people from Jimma town and the surrounding areas. The pediatrics outpatient department (OPD) that operates from Monday to Friday was serving on average 50 children daily. Its routine function includes management of medical illness, child growth monitoring and nutritional advice; provision of outpatient therapeutic-food programs and provision of initiative HIV counseling and testing services. 

### 2.2. Sampling of Study Population

All children under five attending pediatric outpatient department during the study period and who were seen for either well-child care visits or sick care visits were eligible for the study. Critically ill children and those taking antibiotics were excluded from the study. The required sample size was computed using the general formula for a single population proportion with the following assumptions: prevalence rate of 36% taken from Eldoret, Kenya [17], 95% confidence level and 5% marginal error. Considering 5% for anticipated non-response rate, the total sample size calculated was 372. The study participants were selected using a systematic random sampling method.

### 2.3. Collection of Demographic and Clinical Data

Socio-demographic and clinical data were collected using a pre-tested and standardized questionnaire. The questionnaire contained mainly close ended and few open ended questions on socio-demographic, economic characteristics and house hold environmental factors. Moreover, clinical respiratory illness characteristics specifically pneumonia, otitis media and sinusitis were collected by reviewing the medical records after clinical diagnosis by a general practitioner.

### 2.4. Measurement of Nutritional Status

Anthropometric measurement was taken to assess nutritional status of children. Weight in kilograms was measured using a weighing scale in standing manner for those children who can stand erect and lying down for those cannot stand erect by a trained clinical nurse in OPD of the hospital. The children were weighed wearing light cloths and without shoes. The weighing scale was calibrated to zero before taking every measurement. Bilateral oedema was determined by applying finger pressure on both feet for three seconds. If a shallow print persisted on the both feet, then the child presented nutritional oedema. Nutritional status was categorized as malnourished or not among children as a predictor factor by using weight for age Z-scores (WAZ). The WAZ was calculated using WHO anthro version 3.2.2 software application [18]. A child was considered as underweight if the corresponding WAZ scores are less than −2 standard deviation (SD) [19]. Malnutrition was defined as below −2 SD score from the median weight-for-age of the reference population according to WHO child growth standards and/or if a child had oedema on both feet [20].

### 2.5. Collection of Nasopharyngeal Swab 

Nasopharyngeal swap was collected per child by gentle insertion of sterile flexible plastic applicator rayon tipped swab (Copan, Brescia, Italy). Collected swabs were placed immediately in 1 mL skim-milk tryptone glucose glycerol (STGG) transport media. The samples were transported to Jimma University, Medical Microbiology laboratory and stored at −20 °C. All swabs were processed within 8 h of collection. 

### 2.6. Identification of S. pneumoniae

*S. pneumoniae* was identified based on the methods outlined and the recommendations given by WHO manual [21]. NP swab-STGG specimens were mixed thoroughly using vortex. A sample of 10 µL were inoculated onto tryptone soya agar base (Oxoid Ltd., Basingstoke, Hampshire, UK) supplemented with 5% sheep blood and 5 µg/mL gentamycin plates (CSPC Ougyi Pharmaceutical, Shijiazuang, China). The inoculums were streaked using sterile wire loop and the streaked plates were incubated into 5–10% CO_2_ atmosphere using candle jar at 37 °C for 24 h. Gram-positive cocci alpha hemolytic colonies on the plates were picked up with sterile wire loop and streaked (sub-cultured) onto tryptone soy agar base supplemented with 5% sheep blood. Optochin disks with 6 mm diameter containing 5 µg of ethylhydrocupreine (Himedia Laboratories, Mumbai, India) were placed aseptically on the streaks of inoculum then plates were incubated in 5–10% CO_2_ atmosphere at 37 °C for 24 h. Optochin-susceptible strains with ≥14 mm in diameter of the zone of inhibition were identified as *S. pneumoniae*; strains with zones of inhibition < 14 mm were subjected to bile solubility test (tube method) using 2% sodium deoxycholate or bile salt (Oxoid Ltd.). The overall flow chart explaining identification algorithm for *S. pneumoniae* isolates was depicted in Figure S1.

### 2.7. Antimicrobial Susceptibility Testing of Isolates 

All isolates of *S. pneumoniae* were tested against tetracycline (TE) 30 µg, trimethoprim-sulfamethoxazole (TMP-SMX) 1.25 + 23.75 µg, oxacillin (OX) 1 µg, chloramphenicol (C) 30 µg, and erythromycin (E) 15 µg antimicrobial disks (Oxoid Ltd) using disk diffusion (modified Kirbye-Bauer) method on Mueller–Hinton agar (Oxoid Ltd.) supplemented with 5% sheep blood [22]. The antimicrobial agents were selected based on the prescription practices in hospital and also Ethiopian standard treatment guideline for the treatment of *S. pneumoniae* infection as well as the susceptibility test type affordability. Pure colonies were taken from plates with fresh pure culture using sterile wire loops, suspended in sterile normal saline. The turbidity of suspension was adjusted comparable to a 0.5 McFarland standard and it was used within 15 min of preparation. A sterile cotton swab was dipped into the adjusted suspension and excess was removed by gentle rotation of the swab against inside wall of the tube. The swab was inoculated evenly over the entire surface of Mueller–Hinton agar supplemented with 5% sheep blood, then the inoculated plates were allowed to air dry. The disks were placed aseptically on the plate using sterile forceps then plates were incubated in a 5–10% CO_2_ atmosphere at 37 °C for 24 h. Finally, inhibition zone diameters were measured to the nearest millimeters from disks using a ruler. *S. pneumoniae*-American Type Culture Collection (ATCC 49,619) was used as a positive control strain on each procedure. The results were interpreted by comparing to cut-off points in the Clinical and Laboratory Standard Institute (CLSI) result interpretive standards [22]. Interpretation of penicillin susceptibility testing were performed using oxacillin (1 µg) disks by comparing the results to the interpretive standards of CLSI [22].

### 2.8. Ethical Approval

The study protocol was reviewed and approved by Ethical Review Committee of College of Health Sciences, Jimma University, Ethiopia (RPGC/469/2014). Informed oral and written consent was obtained from parent or legal guardian of respective child. Any information concerning the children was kept confidential.

### 2.9. Statistical Analysis

Data were entered, cleaned and analyzed using Statistical Package for Social Sciences (SPSS) version 20.0 statistical software (IBM corporation, Chicago, IL, USA). Anthropometric WAZ values were initially calculated using WHO anthro version 3.2.2 software application [18]. Descriptive statistics were used to summarize socio-demographic, carriage rate and susceptibility patterns of isolates. Bivariate and multivariate logistic regression analyses were carried out to identify potential factors of pneumococcal NP carriage. Adjusted odds ratio with the corresponding 95% confidence intervals (CI) was used to measure the association between potential risk factors and NP carriage. Those variables at a cut-off point *p*-value less than 0.25 in bivariate analysis were candidate for multivariate analysis. *p* values less than 0.05 were considered statistically significant. 

## 3. Results

### 3.1. Characteristics of the Study Participants

Of 372 children under five surveyed between June and September 2014, 11children refused to be swabbed. Therefore, a total of 361 children under five attending pediatric outpatient department of Shanan Gibe Hospital were enrolled in this study. Male children, 206 (57.1%), slightly outnumbered female children, 155 (42.9%). Age of children ranged from 2 to 59 months with mean age of 24.5 ± 16.8 months. Nearly half, 172 (47.6%), of the children were with family size of above five persons per household and 258 (71.5%) children had sibling(s) living together (Table 1).

A total of 274 (76%) children had received one does or more of pneumococcal conjugate vaccine (249 fully vaccinated and 25 partially vaccinated). Eighty-two (22.7%) of 361 children were malnourished (below −2 standard deviation score). Of total children enrolled, 110 (34.8%) were found to have respiratory illnesses including 79 (21.9%) children with pneumonia, 19 (5.3%) with sinusitis and 12 (3.3%) with otitis media. The demographical and clinical characteristics of the 361 children included in this study are shown in Table 1.

### 3.2. Nasopharyngeal Carriage Rate of S. pneumoniae

Of 361 children under five tested, 158 (43.8%) were found to carry *S. pneumonia* in their nasopharynx. The difference in pneumococcal carriage between female (45.8%) and male (42.2%) children was not statistically significant. The *S. pneumoniae* NP carriage rate tended to increase with age, from 41.7% in the 2–23 months age group to 48.7% in the 42 months and older age groups, though not statistical significant. Out of 158 children whom were colonized with *S. pneumoniae,* 115 (72.8%) received PCV; 105 were fully vaccinated (received 3 doses of PCV) and 10 were partially vaccinated (received 1 or 2 dose of PCV).

### 3.3. Antimicrobial Susceptibility Patterns of Isolates

Antimicrobial susceptibility patterns were determined for all 158 isolates of *S. pneumoniae* to five antimicrobial agents. Of all isolates, only 34 (21.5%) isolates were susceptible to all of the five antibiotics tested, 39 (24.7%) were resistant to one antimicrobial agent, 57 (36.1%) were resistant to two antimicrobials and 28 (17.7%) of isolates were multidrug resistance (resistant to three or more antimicrobials). 

Highest degree of resistance among the five antibiotics was seen for tetracycline followed by TMP-SMX and penicillin. Eighty-four (53.2%) isolates were resistant to tetracycline: 69 (43.7%) isolates were fully resistant and 15 (9.5%) were intermediately resistant. Sixty (38.0%) isolates were fully resistant and nine (5.7%) were intermediately resistant to TMP-SMX. Complete resistant to penicillin was seen in 57 (36.1%) isolates. The lower rate of resistance was documented for two antimicrobial agents: chloramphenicol (13.3%) and erythromycin (8.9%) (Table 2).

### 3.4. Risk factors for Nasopharyngeal Carriage of S. pneumoniae

Table 3 shows the results of the univariate and multivariate analysis of the potential risk factors for the nasopharyngeal carriage of *S. pneumoniae*. We compared several characteristics between *S. pneumoniae* carriers and non-carriers. On multivariate analysis, we found two factors associated with the increased risk of *S. pneumoniae* in children under five, i.e., living with sibling(s) < 5 years old and malnutrition. Children living with sibling(s) < 5 years old were twice as likely to have increased pneumococcal nasopharyngeal carriage when compared with children who were not living with younger sibling(s) < 5 years (Adjusted Odds Ratio (AOR) = 1.798; 95% CI, 1.169–2.766; *p* value = 0.008). In addition, malnourished children had more than twice as likely to have increased carriage as children who were not malnourished (AOR = 2.065; 95% CI, 1.239–3.443; *p* value = 0.005). 

We found no differences in nasopharyngeal carriage rates by sex, age, place of residence, attending kindergarten, family size, children having one or more siblings, family income, mother educational level, number of rooms in the house, passive smoking, main sources of cooking fuel type, PCV vaccination status, and recent respiratory illness (*p* value > 0.05). For example, out of 249 PCV fully vaccinated and 87 non-vaccinated children, 105 (42.2%) and 43 (49.4%) respectively were carriers of *S. pneumoniae* in their nasopharynx, however with no statistically significant difference (Table 3).

## 4. Discussion

The asymptomatic nasopharyngeal carriage of *S. pneumoniae* is widely prevalent in young children and has been related to the development of disease and the spread of the pathogen in the community. The prevalence of nasopharyngeal carriage of *S. pneumoniae* varies based on age, geographical area, crowding, concomitant respiratory tract illness, nutritional status, and sampling technique. We assessed the prevalence of nasopharyngeal carriage of *S. pneumoniae,* determined their antimicrobial susceptibility to commonly prescribed antibiotics, and identified the risk factors for carrier status among children attending a primary care hospital in Jimma, Southwest Ethiopia.

Several studies in different parts of the world have documented a wide range of nasopharyngeal carriage rates of *S. pneumoniae*. In the present study, higher nasopharyngeal carriage rate of *S. pneumoniae* (43.8%) was found among children aged under five years. Similarly high rates have also been found in other countries, with carriage rates ranging from 39% to 43% [14,23] although lower carriage rates (17–24%) were observed in some previous studies [24]. In contrast, much higher carriage rates were seen in a few African countries: 87.0% in Mozambique [25] and 65.8% in Kilifi, Kenya [26]. The children who were visiting hospital may have had possible unrecognized underlying immunosuppressive conditions in addition to malnutrition that prompted them to seek medical attention; these could be the contributory factors for higher carriage in our finding. This finding implicates that many children carrying the *S. pneumonia* in their nasopharynx have the risk of developing pneumococcal diseases and they become the most important source for horizontal dissemination of the pathogen within the community.

Treatment of infections due to *S. pneumonia* has become a complicated global problem due to antibiotic resistance. In our study, high carriages (53.2%) of tetracycline-resistant pneumococci strains were observed. Tetracycline resistant *S. pneumoniae* in various studies have been reported to range from 25–65% [14,27]. Tetracycline is one the most commonly available antimicrobials in most public and private health facilities. It has been used for the treatment of respiratory tract illnesses (symptoms) without prescription (self-medication) in the community. However, use of tetracycline is reported to cause teeth retardation for children under 8 years old making them less suitable for oral use in children. 

Excessive consumption, inappropriate and overuse of TMP-SMX are possibly the contributory factors for high resistance *of S. pneumoniae* to these antibiotics in our study. TMP-SMX has been extensively used for respiratory tract infections, because of its broad antimicrobial coverage, and synergetic effects, and because it is inexpensive. The pneumococcal nasopharyngeal isolates also demonstrated resistance to other antimicrobial agents like penicillin, chloramphenicol, and erythromycin. In the present study, 36% of isolates were resistant to penicillin. Resistance to penicillin is reported to be a surrogate marker for the presence of a multidrug-resistant phenotype. Strains with increased resistance to penicillin are usually cross-resistant to other antibiotics [28].

Multidrug-resistant *S. pneumonie* is increasingly being reported from many parts of the globe [29]. In our study, 17.7% of isolates were multidrug-resistant. The majority of multidrug resistant isolates showed a profile of resistance to tetracycline, penicillin and TMP-SMX. In most developing countries like Ethiopia, there is uncontrolled availability of antimicrobial agents and it is a common practice that antimicrobials can be illegally purchased without prescription. This has led to their misuse by the public and, thus contributed to the emergence and spread of antimicrobial resistance in the community [28]. The increased rate of resistance to these antimicrobial agents could be associated with their widespread use for treatment of various infections in the setting, because of their administration convenience, relative cost effectiveness, and easy availability in the community and healthcare facilities.

Previous studies revealed that different factors had been associated with an increase in NP carriage of *S. pneumoniae.* Factors such as young age [14,25,30], family size more than 5 people [31], having at least one sibling(s) [32], number of rooms in the house [14], exposure to tobacco and cooking fuel smoke [30,33], and underlying diseases [14,31,34] were among factors associated with NP carriage rate as reported by previous studies. In our current study, none of the aforementioned characteristics were found to be risk factors for nasopharyngeal carriage of *S. pneumoniae.* The reason for the absence of association in the current study could be partly due to variation in geographical and socioeconomic characters compared to the previous studies.

In the present study, children who had been living together with sibling(s) <5 years old were two times more likely to be colonized with *S. pneumoniae* than those who did not have any younger sibling(s) <5 years old. This finding is concordant with the results reported from Semarang, Indonesia [30] and from Gondar, Ethiopia [14]. This can be explained by the fact that, younger sibling(s) <5 years old have immunological immaturity and carry *S. pneumoniae* in their nasopharynx niche in high magnitude. Living together with these younger children promotes exposure with them and makes it easy to acquire *S. pneumonia* colonization. We also identified malnutrition as an independent risk factor for *S. pneumoniae* colonization as observed in previous studies from Eñepa, India [34]. Malnutrition can impair the immune system and lead to persistent and recurrent colonization with pneumococci. Despite good pneumococcal vaccine coverage in Ethiopia, *S. pneumonia* carriage rate showed no significant difference between the vaccinated and non-vaccinated children. This might be due to the replacement of serotypes that were already in circulation or not included in the PCV and capsular switching of *S. pneumoniae* by recombination such that the immune pressure by the PCV vaccine selected for the serotypes is not included in it.

The findings of this study should be interpreted with the consideration of the following limitations. First, we failed to serotype the isolates found in this study. We were unable to comment on the proportion of invasive serotypes covered by the current PCV vaccine. Secondly, penicillin resistance was determined by the disc diffusion method. Although the disc diffusion test is a good screening test, is not sufficient to classify strains as penicillin-resistant. Thirdly, this study was hospital-based and may not reflect the actual prevalence of *S. pneumoniae* carriage rate in the community. 

## 5. Conclusions

There is high nasopharyngeal carriage of *S. pneumoniae* among young children in Jimma and the surrounding area, where children could be the most important reservoir for horizontal dissemination of the pathogen within the community. High nasopharyngeal carriage of tetracycline and TMP-SMX resistant *S. pneumoniae* was documented in Southwest Ethiopia. Nasopharyngeal carriage is independently predicted by living with sibling(s) <5 years old and malnutrition. There should be a strategy to prevent *S. pneumoniae* nasopharyngeal colonization and identify the appropriate antibiotic to the individual child. Further research is warranted regarding the distribution of pneumococcal serotypes and resistance for each region to accurately evaluate the effectiveness of the vaccine.

## Figures and Tables

**Table 1:** Socio-demographic and clinical characteristics of children under five included in this study (*n* = 361).

Characteristics Study Participants		*n* (%)
Sex	Male	206 (57.1)
	Female	155 (42.9)
Age in months	2–23	192 (53.2)
	24–41	93 (25.8)
	42–59	76 (21.0)
Place of residence	Urban	284 (78.7)
	Rural	77 (21.3)
Attending kindergarten/school	Yes	16 (4.4)
	No	345 (95.6)
Family size	<5	189 (52.4)
	≥5	172 (47.6)
Having sibling(s) (at least 1) in house	Yes	258 (71.5)
	No	103 (28.5)
Age of sibling(s) < 5 years	Yes	164 (45.4)
	No	197 (54.6)
Age of sibling(s) ≥ 5 years	Yes	194 (53.7)
	No	168 (46.3)
Bed sharing with parent/guardian	Yes	294 (81.4)
	No	67 (18.6)
Number of room(s) in the house	1	90 (24.9)
	≥2	271 (75.1)
PCV immunization status	Fully vaccinated *	249 (69)
	Partially vaccinated **	25 (6.9)
	None vaccinated	87 (24.1)
Pneumonia	Yes	79 (21.9)
	No	282 (78.1)
Otitis media	Yes	12 (3.3)
	No	349 (96.7)
Sinusitis	Yes	19 (5.3)
	No	342 (94.7)

* vaccinated with 3 doses of pneumococcal conjugate vaccine (PCV); ** vaccinated with 1 or 2 doses of PCV.

**Table 2:** Antimicrobial susceptibility patterns of *Streptococcus pneumoniae* isolates (*n* = 158).

Antimicrobial Agents	Susceptibility Pattern		
	Resistant	Intermediate	Susceptible
	*n* (%)	*n* (%)	*n* (%)
Tetracycline	69 (43.7)	15 (9.5)	74 (46.8)
TMP-SMX	60 (38.0)	9 (5.7)	89 (56.3)
Penicillin	57 (36.1)	0 (0)	101 (63.9)
Chloramphenicol	21 (13.3)	0 (0)	137 (86.7)
Erythromycin	9 (5.7)	5 (3.2)	144 (91.1)

TMP-SMX: Trimethoprim-sulfamethoxazole.

**Table 3:** Univariate and multivariate analysis of the variables potentially associated with the nasopharyngeal carriage of *S. pneumoniae.*

Characteristics		Non–Carrier	Carrier	COR (95% CI)	*p*-Value	AOR (95%CI)	*p*-Value
		*n* (%)	*n* (%)				
**Sex**	Female	84 (54.2)	71 (45.8)	1.16 (0.76–1.58)	0.50		
	Male	119 (57.8)	87 (42.2)	1			
**Age in months**	2–23	112 (58.3)	80 (41.7)	0.75 (0.44–1.28)	0.29		
	24–41	52 (55.9)	41 (44.1)	0.83 (0.45–1.53)	0.55		
	42–59	39 (51.3)	37 (48.7)	1			
**Residence**	Rural	39 (50.6)	38 (49.4)	1.33 (0.80–2.21)			
	Urban	164 (57.7)	120 (42.3)	1			
**Attending school**	Yes	6 (37.5)	10 (62.5)	2.22 (0.80–6.24)	0.13	2.12 (0.72–6.28)	0.18
	No	197 (57.1)	148 (42.9)	1		1	
**Family size**	≥5	98 (57.0)	74 (43.0)	0.94 (0.62–1.43)	0.79		
	<5	105 (55.6)	84 (44.4)	1			
**Having sibling (s)**	Yes	136 (52.7)	122 (47.3)	1.61 (1.01–2.55)	0.03	1.16 (0.66–2.03)	0.61
	No	67 (65.0)	36 (35.0)	1		1	
**Sibling(s) < 5 years old**	Yes	78 (47.6)	86 (52.4)	1.96 (1.28–2.98)	0.002	1.80 (1.17–2.77)	0.008
	No	125 (63.5)	72 (36.5)	1		1	
**Sibling(s) ≥ 5 years old**	Yes	113 (58.2)	81 (41.8)	0.82 (0.54–1.25)	0.35		
	No	90 (53.9)	77 (46.1)	1			
**Bed sharing with parent**	Yes	166 (56.5)	128 (43.5)	0.95 (0.56–1.62)	0.85		
	No	37 (55.2)	30 (44.8)	1			
**Number of room(s) in the house**	1	50 (55.6)	40 (44.4)	1.04 (0.64–1.68)	0.88		
	≥2	153 (56.5)	118 (43.5)	1			
**PCV Vaccination status**	Fully *	144 (57.8)	105 (42.2)	0.75 (0.46–1.22)	0.24	1.07 (0.60–1.89)	0.82
	Partially **	15 (60.0)	10 (40.0)	0.68 (0.28–1.68)	0.40	1.06 (0.40–2.83)	0.90
	None ***	44 (50.6)	43 (49.4)	1		1	
**Malnutrition**	Yes	34 (41.5)	48 (58.5)	2.20 (1.33–3.63)	0.002	2.07 (1.24–3.44)	0.005
	No	169 (60.6)	110 (39.4)	1		1	
**Pneumonia**	Yes	42 (53.2)	37 (46.8)	1.17 (0.71–1.93)	0.53		
	No	161 (57.1)	121 (42.9)	1			
**Sinusitis**	Yes	8 (42.1)	11 (57.9)	1.82 (0.72–4.65)	0.208	2.02 (0.77–5.30)	0.15
	No	195 (57.0)	147 (43.0)	1		1	
**Otitis media**	Yes	4 (33.3)	8 (67.7)	2.65 (0.78–8.98)	0.12	3.15 (0.09–10.9)	0.07
	No	199 (57.0)	150 (43.0)	1		1	

* vaccinated with 3 doses; ** vaccinated with 1 or 2 doses; *** unvaccinated; COR: crude odd ratio; AOR: adjusted odds ratio; CI: 95% confidence interval.

## Supplementary Materials

The following are available online at http://www.mdpi.com/2227-9067/4/4/27/s1, Figure S1: Flow chart explaining the identification algorithm for *S. pneumoniae isolates*.
