# Supplementary material for: Nasopharyngeal Carriage and Antimicrobial Susceptibility Patterns of Streptococcus pneumoniae among Children under Five in Southwest Ethiopia
Source: Children (Basel). 2017 Apr 19;4(4):27. doi: 10.3390/children4040027 (PMC5406686; doi:10.3390/children4040027)
Supplement: Supplementary file 1 [file children-04-00027-s001.pdf]

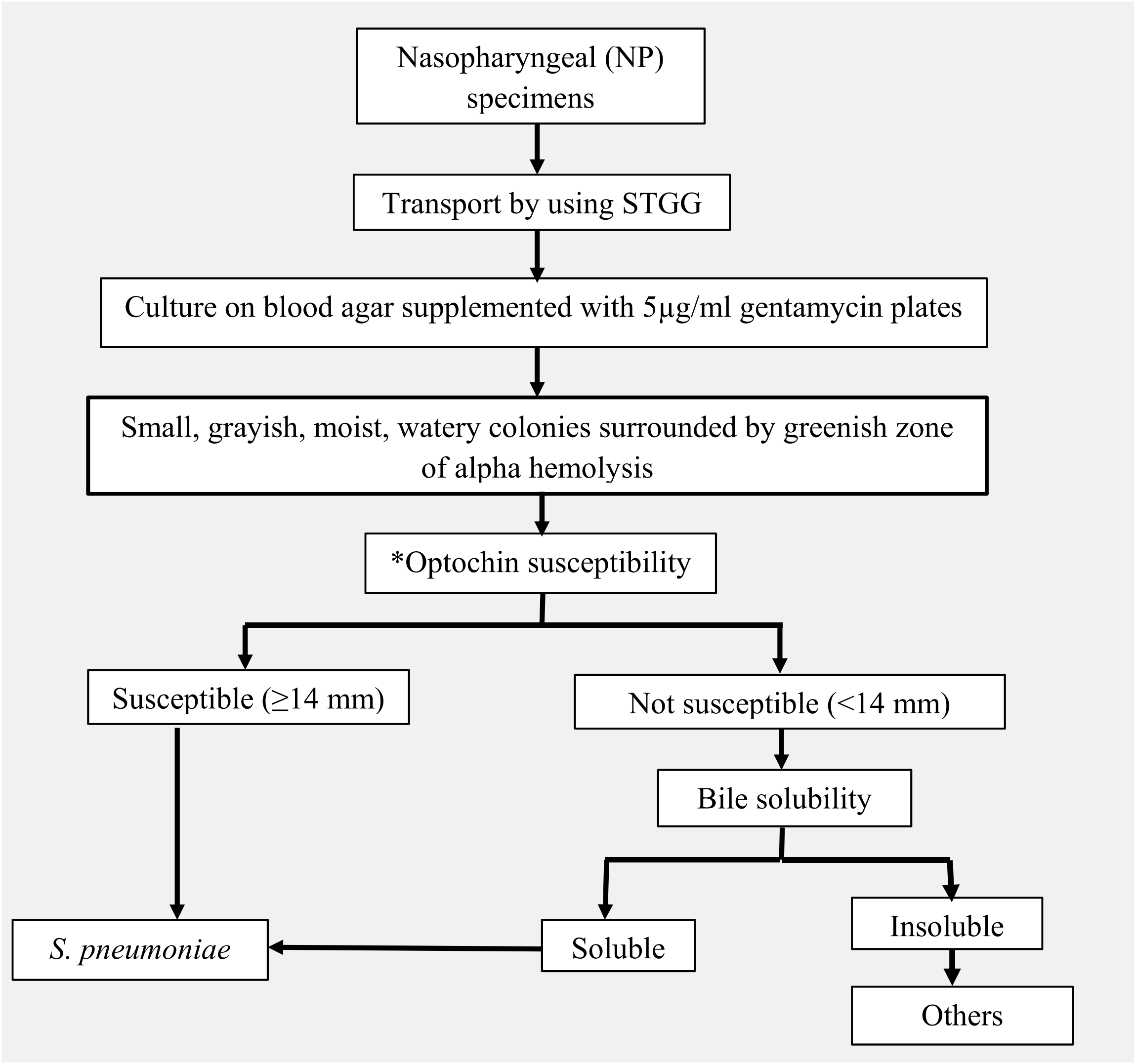

Figure S1: Flow chart explaining the identification algorithm for *S. pneumoniae isolates.*

*Disks with 6 mm diameter and 5µg concentration
